# Supplementary material for: Coproducing healthcare service improvement for people with common mental health disorders including psychotic experiences: a study protocol of a multiperspective qualitative study
Source: BMJ Open. 2018 Nov 8;8(11):e026064. doi: 10.1136/bmjopen-2018-026064 (PMC6231599; doi:10.1136/bmjopen-2018-026064)
Supplement: Author's manuscript [file bmjopen-2018-026064.draft_revisions.pdf]

BMJ Open is committed to open peer review. As part of this commitment we make the peer review history of every article we publish publicly available.

When an article is published we post the peer reviewers' comments and the authors' responses online. We also post the versions of the paper that were used during peer review. These are the versions that the peer review comments apply to.

The versions of the paper that follow are the versions that were submitted during the peer review process. They are not the versions of record or the final published versions. They should not be cited or distributed as the published version of this manuscript.

BMJ Open is an open access journal and the full, final, typeset and author-corrected version of record of the manuscript is available on our site with no access controls, subscription charges or pay-per-view fees (http://bmjopen.bmj.com).

If you have any questions on BMJ Open's open peer review process please email info.bmjopen@bmj.com

BMJ Open

Co-producing healthcare service improvement for people with common mental health disorders including psychotic experiences: a study protocol of a multi-perspective qualitative study.

Journal:	*BMJ Open*
Manuscript ID	bmjopen-2018-026064
Article Type:	Protocol
Date Submitted by the Author:	15-Aug-2018

Complete List of Authors:	Georgiadis, Alexandros; University of Cambridge , THIS Institute (The Healthcare Improvement Studies Institute), Primary Care Unit, Department of Public Health and Primary Care<br>Duschinsky, Robbie; University of Cambridge Primary Care Unit, Applied Social Science Group, Department of Public Health and Primary Care<br>Perez, Jesus; Cambridgeshire & Peterborough NHS Foundation Trust, CAMEO<br>Jones, Peter; University of Cambridge, Department of Psychiatry<br>Russo, Debra; University of Cambridge Department of Psychiatry<br>Knight, Clare; University of Cambridge Department of Psychiatry<br>Soneson, Emma ; University of Cambridge Department of Psychiatry<br>Dixon-Woods, Mary ; University of Cambridge , THIS Institute (The Healthcare Improvement Studies Institute), Primary Care Unit, Department of Public Health and Primary Care

Keywords:	PRIMARY CARE, QUALITATIVE RESEARCH, MENTAL HEALTH, Anxiety disorders < PSYCHIATRY, Depression & mood disorders < PSYCHIATRY

SCHOLARONE™ Manuscripts

Co-producing healthcare service improvement for people with common mental health disorders including psychotic experiences: a study protocol of a multi-perspective qualitative study.

Georgiadis A,[1] Duschinsky R,[2] Perez J,[3] Jones P,[4] Debra R,[5] Knight C,[6] Soneson E,[7] Dixon-Woods M.[8]

[1] Alexandros Georgiadis, THIS Institute (The Healthcare Improvement Studies Institute), Primary Care Unit, Department of Public Health and Primary Care, University of Cambridge. Cambridge Biomedical Campus, Clifford Allbutt Building, Cambridge, CB2 0AH, UK. Email: alex.georgiadis@thisinstitute.cam.ac.uk, tel: 01223761601 (corresponding author)

[2] Robbie Duschinsky, Applied Social Science Group, Primary Care Unit, Department of Public Health and Primary Care, University of Cambridge. Cambridge, UK.

[3] Jesus Perez, Cambridgeshire and Peterborough Foundation Trust, Cambridge, UK.

[4] Peter Jones, Department of Psychiatry, University of Cambridge, Cambridge, UK.

[5] Debra Russo, Department of Psychiatry, University of Cambridge, UK.

[6] Clare Knight, Department of Psychiatry, University of Cambridge, UK.

[7] Emma Soneson, Department of Psychiatry, University of Cambridge, UK.

[8] Mary Dixon-Woods, THIS Institute (The Healthcare Improvement Studies Institute), Primary Care Unit, Department of Public Health and Primary Care, University of Cambridge.

ABSTRACT

Introduction

Some people who have common mental health disorders, such as depression and anxiety, also have some psychotic experiences. These individuals may experience a treatment gap: their symptoms neither reach the increasingly high threshold for secondary care, nor do they receive full benefit from current interventions offered by the Improving Access to Psychological Therapies (IAPT) programme. The result may be poorer clinical and functional outcomes. A new talking therapy could potentially benefit this group. Informed by principles of coproduction, this study will seek the views of service users and staff to inform the design and development of such a therapy.

Methods and analysis

Semi-structured interviews will be conducted with IAPT service users, therapists and managers based in three different geographical areas in England. Our sample will include (a) approximately 15 service users who will be receiving therapy or will have completed therapy at the time of recruitment, (b) approximately 15 service users who initiated treatment but withdrew, (c) approximately 15 therapists each with at least four months experience in a Step-3 IAPT setting, and (d) three IAPT managers. Data analysis will be based on the constant comparative method.

Ethics and dissemination

For peer review only - http://bmjopen.bmj.com/site/about/guidelines.xhtml

The study has been approved by the London Harrow Research Ethics Committee (reference: 18/LO/0642), and all NHS Trusts have granted permissions to conduct the study. Findings will be published in peer-reviewed academic journals, and presented at academic conferences. We will also produce a "digest" summary of the findings, which will be accessible, visual, and freely available.

Strengths and limitations of the study

- This study will address the needs of a currently under-served group: people with a common mental disorder who also have psychotic experiences.
- The multi-perspective approach will provide insights into limitations of current treatments and inform the development of a new, more targeted talking therapy.
- The study will not include real-time observations of therapeutic encounters which might generate more insight into how service users and therapists interact and co-construct care.

Keywords: mental health; depression; anxiety; psychosis; primary care.

For peer review only

INTRODUCTION

Some people who have common mental health disorders, such as depression and anxiety, also have some psychotic experiences, such as attenuated paranoia or voice hallucinations. [1-7] These experiences are likely indicators of severity of mental distress and associated trauma. For example, people with psychotic experiences are at least seven times less likely to reach remission of depressive symptoms, are at increased risk of self-harm, [1-5] and show poorer response to standard psychological treatments for depression and anxiety, even in combination with pharmacotherapy. [8-10] Despite the clinical relevance of psychotic experiences in anxiety and depression, and the evidence that psychosis, depression and anxiety may share common aetiologies, [11-18] current psychiatric diagnostic classifications do not adequately acknowledge the presence of psychotic experiences in common mental disorders. This has important consequences, not least because psychiatric diagnostic classification systems are used to configure how mental health services are organised in England, which in turn impacts on how those services are accessed and used.

The Improving Access to Psychological Therapies (IAPT) programme in England is particularly important for addressing common mental disorders. IAPT provides steps 2 and 3 in the National Institute for Clinical Excellence (NICE) four-step approach to the treatment of common mental disorders.[19] Cognitive behavioural therapy (CBT) is the predominant, but not the only approach adopted by these services, particularly for depression where there is a wider range of recommended treatments (counselling, couples therapy, interpersonal therapy, brief psychodynamic therapy, and CBT).[20] At step 2, psychological wellbeing practitioners provide referral advice and CBT treatments to people with persistent sub-threshold symptoms and mild to moderate common mental disorders. At step 3, high intensity therapists treat people with moderate to severe common mental health disorders and those who do not show sufficient improvement in response to initial interventions at step 2.

For peer review only - http://bmjopen.bmj.com/site/about/guidelines.xhtml

All IAPT services routinely collect patient-reported outcomes measures. The Patient Health Questionnaire (PHQ-9) [21] and Generalised Anxiety Disorder (GAD-7) [22] measures for depression and anxiety respectively used at baseline and following each treatment session allows the calculation of ‘Reliable Recovery’. This is judged to occur if a person scores below the clinical threshold on PHQ-9 (score ≤10) and GAD-7 (score ≤8) post-treatment, and also shows a significant improvement in their condition. [23] Psychotic experiences are not currently routinely measured in IAPT services, although a recent study has shown that approximately 30% of people treated in IAPT services had psychotic experiences.[24] Reliable recovery remains at around 48% of the IAPT population as a whole, [23] but those with a common mental disorder and psychotic experiences may be much less likely to recover,[24] again suggesting greater severity of illness.

People with a common mental disorder including psychotic experiences thus may face a substantial gap in services; their symptoms do not reach the increasingly high threshold for secondary care [25] but the standard cognitive behavioural therapies used in IAPT do not target psychotic experiences nor the trauma histories with which they are often associated. [8-10] Focusing on mood disturbance alone, as IAPT services currently do, is likely to result in psychotic experiences being left untreated, and could contribute to poorer clinical and functional outcomes.

Just as the condition itself (common mental disorder with psychotic experiences) is scattered across different chapters of DSM-5, evidence-based psychological treatments are scattered between different service settings that are inadequate to assess or treat the condition in a holistic manner. By reconfiguring and assembling such treatments into a new talking therapy for use in the low stigma setting of IAPT services, the prospects of recovery might be greatly improved. To maximise effectiveness and applicability within the IAPT setting, the new talking therapy should be grounded in a thorough understanding of the priorities and

For peer review only - http://bmjopen.bmj.com/site/about/guidelines.xhtml

views of IAPT service-users, therapists and managers, and should be based on principles of co-production.

Co-production: a collaborative approach to healthcare improvement

Based on relationships of reciprocity and mutuality among citizens, service users and clinicians, co-production seeks to improve health and support the development of user-led, people-centred health services. Viewing users as active agents and implies an asset-based model, in which narratives and experiences of illness, services and treatment contribute to health research, service improvement and knowledge, it is firmly rooted in the principle that bringing together clinical, lay, and biomedical knowledge allows new forms of values, knowledge and social relations to emerge. Co-production frameworks are increasingly used as a means to guide the reform and improve healthcare services, [26-28] with the NHS England Five Year Forward View for Mental Health specifying that co-production between managers, clinicians, and service users should be at the heart of service design. Accordingly, the design and execution of our investigation will be based on the involvement of key stakeholders to facilitate the identification and in-depth examination of key priorities for improvement.

The study

The proposed study is part of a wider research programme grant led by the Department of Psychiatry, University of Cambridge, and funded by the National Institute of Health Research (NIHR Programme Grant for Applied Health Research RP-PG-0616-20003). As part of this wider research programme, the proposed study is expected to inform the design and development of a new talking therapy that aims to improve the prospects of recovery for people with a common mental disorder including psychotic experiences who are attending IAPT services.

For peer review only - http://bmjopen.bmj.com/site/about/guidelines.xhtml

Aims and objectives

We will conduct a qualitative study to explore how IAPT users, therapists, and managers perceive the current treatment of people with a common mental disorder including psychotic experiences who are attending Step-3 IAPT services. This work will seek to identify the challenges and positive experiences participants have had in providing or receiving care, and their priorities for its improvement. The aims of the proposed study are to:

1) **Produce an in-depth account of both the challenges and positive experiences reported by IAPT users with a common mental disorder including psychotic experiences in receiving care from current Step-3 IAPT services,** including their views on their therapist(s), the talking therapy they were offered, how it was implemented, how far they saw it as effective in addressing their mental health needs, and their ideas, values and priorities for the development of a new talking treatment and for improving therapists' skills in treating service users with a common mental disorder including psychotic experiences.

2) **Produce an in-depth account of both the challenges and positive experiences of IAPT therapists in delivering care for IAPT users with a common mental disorder including psychotic experiences,** including therapists' understanding of the term "common mental disorder including psychotic experiences", and their views on the value, role and effectiveness of the talking therapy they currently use and of clinical skills in addressing such manifestations, as well as their ideas, values and priorities for the development of a new talking treatment and for improving therapists' skills in treating service users with such experiences.

3) **Produce an in-depth account of IAPT managers' experiences in delivering and supporting the delivery of care for IAPT users with a common mental disorder including psychotic experiences**, including managers' views on the current pathways of care

For peer review only - http://bmjopen.bmj.com/site/about/guidelines.xhtml

and their effectiveness, their impact on meeting performance and recovery targets, as well as managers' ideas, values, and priorities for the development of a new talking treatment and for improving therapists' skills in treating service users with common mental disorders including psychotic experiences.

METHODS AND ANALYSIS

Study design

The study will conduct semi-structured interviews to explore the views of IAPT service users and staff.

Recruitment, sampling, and selection of service users for interview

The proposed study will take place in three IAPT provider sites based in the East and South East England. We aim to recruit up to approximately 30 service users who meet our criteria for a common mental disorder including psychotic experiences from across the participating IAPT provider sites (see Tables 1 and 2). Around half (n=15) will be in therapy (at least four sessions) or will have completed therapy at the time of recruitment; the other half will have initiated treatment but withdrawn. We believe that this number will be sufficient to achieve theoretical saturation [29], where no further themes emerge as further participants are included, but we will monitor this carefully. We will use quota sampling to ensure diversity of gender and ethnicity, by matching the proportion of subgroups to the population (e.g. roughly half of the sample will be female participants). We will then choose potential participants by adhering to the subgroup population proportion.

Sampling

The research team has liaised and worked collaboratively with IAPT managers and therapists from the participating IAPT provider sites to agree on the most convenient procedures for service user recruitment, which will minimise the effort required from IAPT staff whilst maximising the likelihood of reaching the above recruitment targets. The

For peer review only - http://bmjopen.bmj.com/site/about/guidelines.xhtml

proposed study will focus on adult female and male service users, as people below 18 years old have different mental health care structures and service delivery within IAPT and are not the focus of our study. Non-English speakers will be included.

We will employ a two-step method to help us ensure that we identify, approach and recruit the right sample while reducing the risk of causing unnecessary distress to service users. First, in addition to assessment measures currently used for depression (PHQ-9) and anxiety (GAD-7), the participating IAPT provider sites administer the shortened version of the Community Assessment of Psychic Experiences questionnaire (CAPE-P15) [30] as part of a service evaluation to identify the prevalence and impact of psychotic experiences in their current caseloads. All service-users attending step-3 IAPT services are offered the opportunity to complete the self-reported CAPE-P15 questionnaire once during the course of their treatment.

The CAPE-P15 is a 15-item, self-report measure of experiences that are similar to positive psychotic symptoms, such as perceptual abnormalities, persecutory ideation, and bizarre experiences. It measures the frequency (ranging from 1-never to 4 – nearly always) and level of distress (ranging from 1-not distressed to 4-very distressed) that each psychotic experience causes to the individual. To account for non-response to any items, scores are weighted for the number of valid answers. The weighted score is the sum score divided by the amount of items completed (i.e. the mean of completed items). Higher scores indicate a higher frequency of psychotic experiences and an increased level of distress in relation to these experiences. A cut-off point ≥1.47 for frequency and a cut-off point of ≥1.47 for distress has been useful for detecting people who have psychotic experiences. [31] Service users who have scored in ≥1.47 for both frequency and distress in CAPE-P15 will be considered as cases, and therefore, eligible to participate in our study.

For peer review only - http://bmjopen.bmj.com/site/about/guidelines.xhtml

For peer review only

Second, at the end of the CAPE-P15 we have added a query where we ask service users to indicate, by ticking a box, whether they are interested in taking part in the proposed study and discuss their experiences of using step-3 IAPT services. From amongst those service users with scores of ≥1.47 for both frequency and distress on CAPE-P15 we will approach a proportion of those service users (following the quota sampling method) who indicated in the CAPE-P15 questionnaire that they would like to participate in the proposed study. We will also include service users who have indicated in their IAPT registration form that they want to take part in research projects. To ensure confidentiality, we will use service users' IAPT numbers (unique identification numbers, similar to NHS numbers, but exclusively used within IAPT services). This approach will allow us to approach the right sample and minimise the risk of unduly stressing potential participants, as well as ensure that service users' data and confidential information are protected.

Table 1: Inclusion and exclusion criteria for people who are in therapy or have completed their therapy

Inclusion	Exclusion
Male or Female<br>18 years of age or over<br>PHQ-9 score ≥10 and/or GAD-7 score ≥8 (at any point during the therapy)<br>CAPE-P15 scores ≥1.47 for frequency and ≥1.47 for distress<br>Expressed an interest in participating in research either via the CAPE-P15 or IAPT registration form<br>Have completed or attended at least 4 therapy sessions at the time of recruitment	PHQ-9 score ≤10 and/or GAD-7 score ≤8<br>CAPE-P15 scores ≤1.47 for frequency and ≤1.47 for distress<br>Have not expressed an interest in participating in research neither in CAPE-P15 not IAPT registration form<br>Have attended fewer than 4 therapy sessions at the time of recruitment

Table 2: Inclusion and exclusion criteria for people who initiated treatment but withdrawn

Inclusion	Exclusion
Male or Female<br>18 years of age or over<br>PHQ-9 score ≥10 and/or GAD-7 score ≥8 (at any point during the therapy)<br>CAPE-P15 scores ≥1.47 for frequency and	PHQ-9 score ≤10 and/or GAD-7 score ≤8<br>CAPE-P15 scores ≤1.47 for frequency and ≤1.47 for distress<br>Have not expressed an interest in participating in research neither in CAPE-

For peer review only - http://bmjopen.bmj.com/site/about/guidelines.xhtml

≥1.47 for distress<br>Expressed an interest in participating in research either via the CAPE-P15 or IAPT registration form<br>Initiated treatment but withdrawn	P15 nor IAPT registration form

Data collection

Semi-structured interviews will be conducted using a topic guide that is co-produced with our service user advisory group, and that is informed by a review of relevant literature. Members of our service-user advisory group and members of the research team developed the topic guide, and in so doing sought to minimize the risk of unduly stressing participants through content and delivery. The topic guide aims to elicit participants' views on the identification and treatment of people with a common mental disorder including psychotic experiences attending step-3 IAPT services. More specifically, it asks participants to discuss relevant aspects of their personal and life stories, including how past negative life events contributed to their mental health difficulties, and their positive and negative experiences of receiving care from IAPT services. The researcher will explore service users' views on what improvements might be made at the levels of the therapist and talking treatment. The interview will also examine how such improvements should be implemented and prioritized to improve the care that step-3 IAPT services deliver to people with a common mental disorder including psychotic experiences.

Recruitment, sampling, and selection of IAPT therapists and managers for interview

Our aim is to recruit five IAPT therapists and one manager per IAPT provider. We will present details of the study during team meetings and ask for volunteers, with more than six months experience working with step-3 IAPT service users. We will use a purposive sampling method to ensure diversity of professional experience, gender, and ethnicity.

For peer review only - http://bmjopen.bmj.com/site/about/guidelines.xhtml

Data collection

Semi-structured interviews will be conducted using a topic guide which has been co-produced with an IAPT manager and also informed by a review of the relevant literature. The manager and members of the research team developed the topic guide collaboratively, with the aim to develop interview questions and prompts that covered the full scope of topics pertinent to the scope of the study. The topic guide aims to elicit participants' views on the identification and treatment of people with a common mental disorder including psychotic experiences attending step-3 IAPT services. More specifically, the topic guide will ask participants to discuss their positive and negative experiences of delivering care to service users with a common mental disorder including psychotic experiences, the talking therapies they use to treat and care for these users, and their reflections regarding effectiveness. The researcher will also explore the types of knowledge that participants draw upon to provide care and support these service users. Additionally, the interview will ask about therapists' and managers' ideas, values and priorities for the development of a new talking treatment and for improving therapists' skills in treating service users with a common mental disorder including psychotic experiences.

Data analysis

All interview data will be coded by one researcher experienced in qualitative research methods. Although we will not formally test inter-rater reliability, a sample of the collected interview data (n=5) will be double-coded by another researcher. Analysis will be based on the constant comparative method as described by Charmaz. [29] We will draw on the sensitising constructs identified through the literature, but we will remain open to the emergence of other important themes which may be more relevant to the primary care mental health setting. NVivo software (NVivo v11, QSR International, Doncaster, Australia) will be used to facilitate data coding. The combination of the different strands of data (i.e. semi-structured

For peer review only - http://bmjopen.bmj.com/site/about/guidelines.xhtml

interviews with IAPT users, therapists and managers), will be brought together to inform the development of a new talking therapy for people with a common mental disorder including psychotic experiences.

Patient and Public Involvement

The McPin Foundation (http://mcpin.org) convened a service user advisory group for the programme. This involves nine people with common mental disorders including psychotic experiences from diverse backgrounds, including ethnic minorities. For this particular work-package, the research team work collaboratively with the service user advisory group to develop the IAPT service user invitation letter, information sheet, and interview topic-guide list. All study participants will receive a digest summary of findings written in lay English language.

ETHICS AND DISSEMINATION

The following permissions have been obtained for this study:

1) NHS Ethics – approval granted by London Harrow Research Ethics Committee (reference: 18/LO/0642);
2) NHS Permissions were granted by all Trusts involved;
3) We registered the study on the UK Clinical Research Network Study Portfolio (reference: 38180)

Ethical considerations

A number of steps will be taken to ensure that the research is conducted ethically, protecting participants' rights and maintaining appropriate confidentiality of the information provided.

For peer review only - http://bmjopen.bmj.com/site/about/guidelines.xhtml

Voluntary nature of participation and right to withdraw

Information sheets for participants, as well as researcher verbal reassurance, will clarify that participation in the study is voluntary. No pressure will be placed on any individual to take part in the research. It will be made clear to participants that their decision to take part or decline to take part in any part of the research will affect neither the care they receive nor their legal rights. Participants will also be informed about their right to withdraw from the study.

Informed consent

All participants will be asked to give their informed consent before any data are collected. Such consent will only be sought after they have been provided with full information about the research and what their participation would involve. Additionally they will be given sufficient time to consider the aims and scope of the study and ask questions. Service users will not be approached about the research if – in the opinion of mental health professionals – (i) they are unable to give informed consent, (ii) their current mental health gives significant cause for concern, and (iii) their mental health may be adversely affected by taking part in the research. The researcher will liaise with the participating IAPT teams to ensure that the potential participants selected to be contacted and invited to participate in an interview do not meet any of the above criteria.

Safety of participants and researcher

Consideration has been given to ways in which taking part in the research might be harmful to participants (in particular service users) and steps will be taken to manage such potential outcomes should they occur. Clinical risk assessments are routinely undertaken within IAPT services to identify during the initial assessment anyone at high risk of harm to themselves or to other people. High risk individuals will be referred on to appropriate services and, accordingly, not be included within our sample. Nevertheless, reflecting on

For peer review only

mental health, traumatic experiences and events, and past health care experiences may cause distress and measures will be taken to ensure the safety of participants. Should participant distress occur during the interviews the researcher will pause the interview, and support the participant to manage the distress and regulate their emotions. The researcher will then check if s/he is still willing to continue with the interview process. If a participant remains distressed, the researcher will ask for permission to contact the participant's GP or mental health worker, whilst, encouraging the participant to seek further support that may be available in their environment. The researcher will have up-to-date contact information for psychiatric emergency services, if required. This will include access to First Response Services and/or Crisis Resolution Home Treatment Teams. These services should be able to advise about how to manage difficult clinical scenarios.

If, during the interview, a participant suggests that they intend to harm themselves or another person, the researcher will ask for the contact details of the participant's GP and inform their GP or mental health worker. The researcher will inform the participant about this action and the reasons for doing this. As stated above, the researcher might also contact psychiatric emergency services in the area. This limit to confidentiality will be explicit in the research information sheet, and it will be explained again before the start of the interview. We believe that the risk of psychological distress to the IAPT therapists and managers will be minimal.

Consideration has also been given to the safety of the researcher who will carry out the interviews. The researcher has attended training to equip him with the skills, awareness and knowledge required for the safe conduct of research in community settings. He is also experienced in carrying out research with vulnerable individuals, including offenders, homeless people, and people with severe mental illness. The researcher will follow the THIS

For peer review only - http://bmjopen.bmj.com/site/about/guidelines.xhtml

Institute's (The Healthcare Improvement Studies Institute, www.thisinstitute.cam.ac.uk) lone worker policy when interviewing people at their homes.

Dissemination

We will report the findings to national and international, multi-disciplinary audiences to inform future research and practice using a variety of methods. We will disseminate original articles in peer-reviewed academic journals, conference presentations, and learning reports. We will also produce a "digest" summary of the findings, which will be accessible, visual, and freely available.

STUDY STATUS

Data collection started in June 2018 and is expected to last 7 months.

DISCUSSION

Improving the prospects of recovery for all people experiencing mental health disorders is a priority in the mental health policy agenda in England. Within this context, this study aims to inform the development of a new talking therapy that will increase the prospects of recovery for people with a common mental disorder including psychotic experiences.

AUTHORS' CONTRIBUTION

Conception or design of the work

Peter Jones, Jesus Perez, Robbie Duschinsky, Mary Dixon-Woods

Drafting the article

Alexandros Georgiadis, Robbie Duschinsky, Mary Dixon-Woods

Critical revision of the article

Alexandros Georgiadis, Robbie Duschinsky, Jesus Perez, Peter Jones, Debra Russo, Clare Knight, Emma Soneson, Mary Dixon-Woods

For peer review only - http://bmjopen.bmj.com/site/about/guidelines.xhtml

Final approval of the version to be published

Alexandros Georgiadis, Robbie Duschinsky, Jesus Perez, Peter Jones, Debra Russo, Clare Knight, Emma Soneson, Mary Dixon-Woods.

ACKOWLEDGMENTS

The authors would like to thank the McPin Foundation, the TYPPEX Lived Experience Advisory Panel, and the participating research sites for helping us to set up this research.

FUNDING STATEMENT

This work was supported by the National Institute for Health Research grant number RP-PG-0616-20003. This work was also supported by Mary Dixon-Wood's Wellcome Trust Investigator award WT09789 and by THIS Institute, which is supported by the Health Foundation. Mary Dixon-Woods and Peter Jones (emeritus) are National Institute for Health Research (NIHR) Senior Investigators.

COMPETING INTERESTS

The authors declare no competing interests.

WORD COUNT: 3454

REFERENCES

1. Hui C, Morcillo C, A RD, et al. Psychiatric morbidity, functioning and quality of life in young people at clinical high risk for psychosis. *Schizophrenia Bulletin* 2013;148(1-3):175-80.
2. Fusar-Poli P, Nelson B, Valmaggia L, et al. Comorbid depressive and anxiety disorders in 509 Individuals with an at-risk mental State: Impact on psychopathology and transition to psychosis. *Schizophrenia Bulletin* 2014;40(1):120-31.
3. Kelleher I, Lynch F, Harley M, et al. Psychotic symptoms in adolescence index risk for suicidal behavior: findings from 2 population-based case-control clinical interview studies. *Archives in General Psychiatry* 2012;69(12):1277-83.
4. Hutton P, Bowe S, Parker S, et al. Prevalence of suicide risk factors in people at ultra-high risk of developing psychosis: a service audit. *Early Intervention in Psychiatry* 2011;5(4):375-80.
5. Granö N, Karjalainen M, Souminen K, et al. Poor functioning ability is associated with high risk of developing psychosis in adolescents *Nordic Juornal of Psychiatry* 2011;65(1):16-21.
6. Salokangas RK, Ruhrmann S, von Reventlow HG, et al. Axis I diagnoses and transition to psychosis in clinical high-risk patients EPOS project: prospective follow-up of 245 clinical high-risk outpatients in four countries. *Schizophrenia Bulletin* 2012;138(2-3):192-97.
7. Wigman JT, van Nierop M, Vollebergh WAM, et al. Evidence that psychotic symptoms are prevalent in disorders of anxiety and depression, impacting on ilness onset, risk,

For peer review only - http://bmjopen.bmj.com/site/about/guidelines.xhtml

and severity - implication for diagnosis and ultra-high risk research. *Schizophrenia Bulletin* 2012;38(2):247-57. doi: 10.1093/schbul/sbr196
8. Wigman J, van Os J, Abidi L, et al. Subclinical psychotic experiences and bipolar spectrum features in depression: association with outcome of psychotherapy. *Psychological Medicine* 2014;44(2):325-36.
9. Perlis RH, Uher R, Ostacher M, et al. Association between bipolar spectrum features and treatment outcomes in outpatients with major depressive disorder. *Archives in General Psychiatry* 2011;68(4):351-60.
10. Are psychotic features in adolescents with depression a predictor of severity? Congress of Psychiatry Medforum; 2015; Wisla, Poland.
11. Kelleher I, Keeley H, Corcoran P, et al. Clinicopathological significance of psychotic experiences in non-psychotic young people: evidence from four population-based studies. *British Journal of Psychiatry* 2012;1:26-32.
12. Varghese D, Scott J, Welham J, et al. Psychotic-like experiences in major depression and anxiety disorders: A population-based survey in young adults. *Schizophrenia Bulletin* 2009;37(2):389-93.
13. Lichtenstein P, Yip BH, Bjork C, et al. Common genetic determinants of schizophrenia and bipolar disorder in Swedish families: a population-based study. *Lancet* 2009;17(373):234-39.
14. van Os J, Linscott R, Myin-Germeys I, et al. A systematic review and meta-analysis of the psychosis continuum: evidence for a psychosis proneness-persistence-impairment model of psychotic disorder. *Psychological Medicine* 2009;39(2):179-85.
15. Nishida A, Tanii H, Nishimura Y, et al. Associations between psychotic-like experiences and mental health status and other psychopathologies among Japanese early teens. *Schizophrenia Bulletin* 2008;99(1-3):125-33.
16. Kraan T, Velthorst E, Smit F, et al. Trauma and recent life events in individuals at ultra high risk for psychosis: Review and meta-analysis. *Schizophrenia Bulletin* 2015;161(2-3):143-49.
17. Russo DA, Stochl J, Painter M, et al. Trauma history characteristics associated with mental states at clinical high risk for psychosis. *Psychiatric Research* 2014;220(1-2):237-44.
18. Kelleher I, Keeley H, Corcoran P, et al. Childhood trauma and psychosis in a prospective cohort study: cause, effect and directionality. *American Journal of Psychiatry* 2013;170(7):734-41.
19. National Collaboration Centre for Mental Health. The Improving Access to Psychological Therapies Manual. London, 2018.
20. Clark DM, Canvin L, Green J, et al. Transparency about the outcomes of mental health services (IAPT approach): an analysis of public data. *Lancet* 2018;391:679-86.
21. Kroenke K, Spitzer RL. The PHQ-9: a new depression diagnostic and severity measure. *Psychiatric Annals* 2002;32:509-15.
22. Spitzer RL, Kroenke K, Williams JB, et al. A brief measure for assessing generalised anxiety disorder: the GAD-7. *Archives of Internal Medicine* 2006;166:1092-97.
23. NHS Digital. Psychological Therapies: Annual report on the use of IAPT services England, further analyses on 2016-17. . Leeds, 2017.
24. Perez J, Russo DA, Stochl J, et al. Common mental disorder including psychotic experiences: Trailblaizing a new recovery pathway within the Improving Access to Psychological Therapies programme in England. *Early Intervention in Psychiatry* 2017:1-8. doi: 10.1111/eip.12434
25. British Medical Association. Breaking down barriers - the challenge of improving mental health outcomes London: British Medical Association, 2017.

For peer review only - http://bmjopen.bmj.com/site/about/guidelines.xhtml

26. Dunston R, Lee A, Boud D, et al. Co-production and health system reform - From re-Imagining to re-Making. *Australian Journal of Public Administration* 2009;68(1):39-52. doi: 10.1111/j.1467-8500.2008.00608.x
27. Batalden M, Batalden P, Margolis P, et al. Coproduction of healthcare service. *BMJ Quality & Safety* 2015;25:509-17.
28. Sabadosa KA, Batalden PB. The interdependent roles of patients, families, and professionals in cyctic fibrosis: a system for the coproduction of healthcare and its improvement. *BMJ Quality & Safety* 2014;23:i90-94.
29. Charmaz K. Constructing Grounded Theory. Thousand Oaks, California: Sage 2014.
30. Capra C, Kavanagh DJ, Hides L, et al. Current CAPE-15: a measure of recent psychotic-like experiences and associated distress. *Early Intervention in Psychiatry* 2013;11(5):411-17. doi: 10.1111/eip.12245
31. Bukenaite A, Stochl J, Mossaheb N, et al. Usefulness of the CAPE-P15 for detecting people at ultra-high risk for psychosis: Psychometric properties and cut-off values. *Schizophrenia Research* 2017;189:6-74.

For peer review only

BMJ Open

Co-producing healthcare service improvement for people with common mental health disorders including psychotic experiences: a study protocol of a multi-perspective qualitative study.

Journal:	*BMJ Open*
Manuscript ID	bmjopen-2018-026064.R1
Article Type:	Protocol
Date Submitted by the Author:	21-Sep-2018
Complete List of Authors:	Georgiadis, Alexandros; University of Cambridge , THIS Institute (The Healthcare Improvement Studies Institute), Primary Care Unit, Department of Public Health and Primary Care<br>Duschinsky, Robbie; University of Cambridge Primary Care Unit, Applied Social Science Group, Department of Public Health and Primary Care<br>Perez, Jesus; Cambridgeshire & Peterborough NHS Foundation Trust, CAMEO<br>Jones, Peter; University of Cambridge, Department of Psychiatry<br>Russo, Debra; University of Cambridge Department of Psychiatry<br>Knight, Clare; University of Cambridge Department of Psychiatry<br>Soneson, Emma ; University of Cambridge Department of Psychiatry<br>Dixon-Woods, Mary ; University of Cambridge , THIS Institute (The Healthcare Improvement Studies Institute), Primary Care Unit, Department of Public Health and Primary Care
**Primary Subject Heading**:	Mental health
Secondary Subject Heading:	Qualitative research, Health services research
Keywords:	PRIMARY CARE, QUALITATIVE RESEARCH, MENTAL HEALTH, Anxiety disorders < PSYCHIATRY, Depression & mood disorders < PSYCHIATRY

Co-producing healthcare service improvement for people with common mental health disorders including psychotic experiences: a study protocol of a multi-perspective qualitative study.

Georgiadis A,[1] Duschinsky R,[2] Perez JB,[3] Jones P,[4] Debra R,[5] Knight C,[6] Soneson E,[7] Dixon-Woods M.[8]

[1] Alexandros Georgiadis, THIS Institute (The Healthcare Improvement Studies Institute), Primary Care Unit, Department of Public Health and Primary Care, University of Cambridge. Cambridge Biomedical Campus, Clifford Allbutt Building, Cambridge, CB2 0AH, UK. Email: ag2000@medschl.cam.ac.uk, tel: 01223761601 (corresponding author)

[2] Robbie Duschinsky, Applied Social Science Group, Primary Care Unit, Department of Public Health and Primary Care, University of Cambridge. Cambridge, UK.

[3] Jesus Perez, Cambridgeshire and Peterborough Foundation Trust, Cambridge, UK.

[4] Peter B. Jones, Department of Psychiatry, University of Cambridge, Cambridge, UK.

[5] Debra Russo, Department of Psychiatry, University of Cambridge, UK.

[6] Clare Knight, Department of Psychiatry, University of Cambridge, UK.

[7] Emma Soneson, Department of Psychiatry, University of Cambridge, UK.

[8] Mary Dixon-Woods, THIS Institute (The Healthcare Improvement Studies Institute), Primary Care Unit, Department of Public Health and Primary Care, University of Cambridge.

For peer review only

ABSTRACT

Introduction

Some people who have common mental health disorders, such as depression and anxiety, also have some psychotic experiences. These individuals may experience a treatment gap: their symptoms neither reach the increasingly high threshold for secondary care, nor do they receive full benefit from current interventions offered by the Improving Access to Psychological Therapies (IAPT) programme. The result may be poorer clinical and functional outcomes. A new talking therapy could potentially benefit this group. Informed by principles of coproduction, this study will seek the views of service users and staff to inform the design and development of such a therapy.

Methods and analysis

Semi-structured interviews will be conducted with IAPT service users, therapists and managers based in three different geographical areas in England. Our sample will include (a) approximately 15 service users who will be receiving therapy or will have completed therapy at the time of recruitment, (b) approximately 15 service users who initiated treatment but withdrew, (c) approximately 15 therapists each with at least four months experience in a Step-3 IAPT setting, and (d) three IAPT managers. Data analysis will be based on the constant comparative method.

For peer review only - http://bmjopen.bmj.com/site/about/guidelines.xhtml

Ethics and dissemination

The study has been approved by the London Harrow Research Ethics Committee (reference: 18/LO/0642), and all NHS Trusts have granted permissions to conduct the study. Findings will be published in peer-reviewed academic journals, and presented at academic conferences. We will also produce a "digest" summary of the findings, which will be accessible, visual, and freely available.

Strengths and limitations of the study

- This study will address the needs of a currently under-served group: people with a common mental disorder who also have psychotic experiences.
- The multi-perspective approach will provide insights into limitations of current treatments and inform the development of a new, more targeted talking therapy.
- The study will not include real-time observations of therapeutic encounters which might generate more insight into how service users and therapists interact and co-construct care.

Keywords: mental health; depression; anxiety; psychosis; primary care.

For peer review only

For peer review only - http://bmjopen.bmj.com/site/about/guidelines.xhtml

INTRODUCTION

Some people who have common mental health disorders, such as depression and anxiety, also have some psychotic experiences, such as attenuated paranoia or voice hallucinations. [1-7] These experiences are likely indicators of severity of mental distress and associated trauma. For example, people with psychotic experiences are at least seven times less likely to reach remission of depressive symptoms, are at increased risk of self-harm, [1-5] and show poorer response to standard psychological treatments for depression and anxiety, even in combination with pharmacotherapy. [8-10] Despite the clinical relevance of psychotic experiences in anxiety and depression, and the evidence that psychosis, depression and anxiety may share common aetiologies, [11-18] current psychiatric diagnostic classifications do not adequately acknowledge the presence of psychotic experiences in common mental disorders. This has important consequences, not least because psychiatric diagnostic classification systems are used to configure how mental health services are organised in England, which in turn impacts on how those services are accessed and used.

The Improving Access to Psychological Therapies (IAPT) programme in England is particularly important for addressing common mental disorders. IAPT provides steps 2 and 3 in the National Institute for Clinical Excellence (NICE) four-step approach to the treatment of common mental disorders.[19] Cognitive behavioural therapy (CBT) is the predominant, but not the only approach adopted by these services, particularly for depression where there is a wider range of recommended treatments (counselling, couples therapy, interpersonal therapy, brief psychodynamic therapy, and CBT).[20] At step 2, psychological wellbeing practitioners provide referral advice and CBT treatments to people with persistent sub-threshold symptoms and mild to moderate common mental disorders. At step 3, high intensity therapists treat people with moderate to severe common mental health disorders and those who do not show sufficient improvement in response to initial interventions at step 2.

For peer review only - http://bmjopen.bmj.com/site/about/guidelines.xhtml

All IAPT services routinely collect patient-reported outcomes measures. The Patient Health Questionnaire (PHQ-9) [21] and Generalised Anxiety Disorder (GAD-7) [22] measures for depression and anxiety respectively used at baseline and following each treatment session allows the calculation of 'Reliable Recovery'. This is judged to occur if a person scores below the clinical threshold on PHQ-9 (score ≤10) and GAD-7 (score ≤8) post-treatment, and also shows a significant improvement in their condition. [23] Psychotic experiences are not currently routinely measured in IAPT services, although a recent study has shown that approximately 30% of people treated in IAPT services had psychotic experiences.[24] Reliable recovery remains at around 48% of the IAPT population as a whole, [23] but those with a common mental disorder and psychotic experiences may be much less likely to recover,[24] again suggesting greater severity of illness.

People with a common mental disorder including psychotic experiences thus may face a substantial gap in services; their symptoms do not reach the increasingly high threshold for secondary care [25] but the standard cognitive behavioural therapies used in IAPT do not target psychotic experiences nor the trauma histories with which they are often associated. [8-10] Focusing on mood disturbance alone, as IAPT services currently do, is likely to result in psychotic experiences being left untreated, and could contribute to poorer clinical and functional outcomes.

Just as the condition itself (common mental disorder with psychotic experiences) is scattered across different chapters of DSM-5, evidence-based psychological treatments are scattered between different service settings that are inadequate to assess or treat the condition in a holistic manner. By reconfiguring and assembling such treatments into a new talking therapy for use in the low stigma setting of IAPT services, the prospects of recovery might be greatly improved. To maximise effectiveness and applicability within the IAPT setting, the new talking therapy should be grounded in a thorough understanding of the priorities and

For peer review only - http://bmjopen.bmj.com/site/about/guidelines.xhtml

views of IAPT service-users, therapists and managers, and should be based on principles of co-production.

Co-production: a collaborative approach to healthcare improvement

Based on relationships of reciprocity and mutuality among citizens, service users and clinicians, co-production seeks to improve health and support the development of user-led, people-centred health services. Viewing users as active agents and employing an asset-based model, in which narratives and experiences of illness, services and treatment contribute to health research, service improvement and knowledge, it is firmly rooted in the principle that bringing together clinical, lay, and biomedical knowledge allows new forms of values, knowledge and social relations to emerge. Co-production frameworks are increasingly used as a means to guide the reform and improvement of mental health services, [26-31] with the NHS England Five Year Forward View for Mental Health specifying that co-production between managers, clinicians, and service users should be at the heart of service design. For example, Memon et al.[32] interviewed 26 adults from black and minority ethnic backgrounds to examine perceived barriers to accessing mental health services, with the aim of informing the development of effective services to improve equity in healthcare. Similarly, Bee et al.[33] explored the views of professionals on how to improve service users and carer involvement in mental health care planning. Accordingly, the design and execution of our investigation will be based on the involvement of key stakeholders to facilitate the identification and in-depth examination of key priorities for improvement.

The study

The proposed study is part of a wider research programme grant led by the Department of Psychiatry, University of Cambridge, and funded by the National Institute of Health Research (NIHR Programme Grant for Applied Health Research RP-PG-0616-20003).

For peer review only - http://bmjopen.bmj.com/site/about/guidelines.xhtml

As part of this wider research programme, the proposed study is expected to inform the design and development of a new talking therapy that aims to improve the prospects of recovery for people with a common mental disorder including psychotic experiences who are attending IAPT services.

Aims and objectives

The study is located within the interpretivist tradition of scientific enquiry exploring individuals' subjective experiences and meaning making processes of social reality. [34] The focus of qualitative methodologies on the meaning and meaning-making processes of social phenomena and events [35] is consistent with the aims of our proposed study, which are to:

1) **Produce an in-depth account of both the challenges and positive experiences reported by IAPT users who have a common mental disorder that includes psychotic experiences in receiving care from current Step-3 IAPT services,** including their views on their therapist(s), the talking therapy they were offered, how it was implemented, how far they saw it as effective in addressing their mental health needs, and their ideas, values and priorities for the development of a new talking treatment and for improving therapists' skills in treating service users with a common mental disorder including psychotic experiences.

2) **Produce an in-depth account of both the challenges and positive experiences of IAPT therapists in delivering care for IAPT users with a common mental disorder including psychotic experiences,** including therapists' understanding of the term "common mental disorder including psychotic experiences", and their views on the value, role and effectiveness of the talking therapy they currently use and of clinical skills in addressing such manifestations, as well as their ideas, values and priorities for the development of a new talking treatment and for improving therapists' skills in treating service users with such experiences.

For peer review only - http://bmjopen.bmj.com/site/about/guidelines.xhtml

3) **Produce an in-depth account of IAPT managers' experiences in delivering and supporting the delivery of care for IAPT users with a common mental disorder including psychotic experiences**, including managers' views on the current pathways of care and their effectiveness, their impact on meeting performance and recovery targets, as well as managers' ideas, values, and priorities for the development of a new talking treatment and for improving therapists' skills in treating service users with common mental disorders including psychotic experiences.

METHODS AND ANALYSIS

Study design

The study will conduct semi-structured interviews to explore the views of IAPT service users and staff.

Recruitment, sampling, and selection of service users for interview

The proposed study will take place in three IAPT provider sites based in the East and South East of England. We aim to recruit up to approximately 30 service users who meet our criteria for a common mental disorder including psychotic experiences from across the participating IAPT provider sites (see Tables 1 and 2). Around half (n=15) will be in therapy (at least four sessions) or will have completed therapy at the time of recruitment; the other half will have initiated treatment but withdrawn. We believe that this number will be sufficient to achieve theoretical saturation [36], whereby no further themes emerge as additional participants are included, but we will monitor this carefully. We will collect demographic data of service users, such as age, gender, and ethnicity. We will use quota sampling to ensure diversity of gender and ethnicity, by matching the proportion of subgroups to the population (e.g. roughly half of the sample will be female participants). We will then choose potential participants by adhering to the subgroup population proportion.

Sampling

For peer review only - http://bmjopen.bmj.com/site/about/guidelines.xhtml

The research team has liaised and worked collaboratively with IAPT managers and therapists from the participating IAPT provider sites to agree on the most convenient procedures for service user recruitment, which will minimise the effort required from IAPT staff whilst maximising the likelihood of reaching the above recruitment targets. The proposed study will focus on adult female and male service users, as people below 18 years old have different mental health care structures and service delivery within IAPT and are not the focus of our study. Non-English speakers will be included.

We will employ a two-step method to help us ensure that we identify, approach and recruit the right sample while reducing the risk of causing unnecessary distress to service users. First, in addition to assessment measures currently used for depression (PHQ-9) and anxiety (GAD-7), the participating IAPT provider sites administer the shortened version of the Community Assessment of Psychic Experiences questionnaire (CAPE-P15) [37] as part of a service evaluation to identify the prevalence and impact of psychotic experiences in their current caseloads. All service-users attending step-3 IAPT services are offered the opportunity to complete the self-reported CAPE-P15 questionnaire once during the course of their treatment.

The CAPE-P15 is a 15-item, self-report measure of experiences that are similar to positive psychotic symptoms, such as perceptual abnormalities, persecutory ideation, and bizarre experiences. It measures the frequency (ranging from 1-never to 4 – nearly always) and level of distress (ranging from 1-not distressed to 4-very distressed) that each psychotic experience causes to the individual. To account for non-response to any items, scores are weighted for the number of valid answers. The weighted score is the sum score divided by the amount of items completed (i.e. the mean of completed items). Higher scores indicate a higher frequency of psychotic experiences and an increased level of distress in relation to these experiences. A cut-off point ≥1.47 for frequency and a cut-off point of ≥1.47 for

For peer review only - http://bmjopen.bmj.com/site/about/guidelines.xhtml

distress has been useful for detecting people who have psychotic experiences. [38] Service users who have scored in ≥1.47 for both frequency and distress in CAPE-P15 will be considered as cases, and therefore, eligible to participate in our study.

Second, at the end of the CAPE-P15 we have added a query where we ask service users to indicate, by ticking a box, whether they are interested in taking part in the proposed study and discuss their experiences of using step-3 IAPT services. From amongst those service users with scores of ≥1.47 for both frequency and distress on CAPE-P15 we will approach a proportion of those service users (following the quota sampling method) who indicated in the CAPE-P15 questionnaire that they would like to participate in the proposed study. We will also include service users who have indicated in their IAPT registration form that they want to take part in research projects. To ensure confidentiality, we will use service users' IAPT numbers (unique identification numbers, similar to NHS numbers, but exclusively used within IAPT services). This approach will allow us to approach the right sample and minimise the risk of unduly stressing potential participants, as well as ensure that service users' data and confidential information are protected.

Table 1: Inclusion and exclusion criteria for people who are in therapy or have completed their therapy

Inclusion	Exclusion
Male or Female<br>18 years of age or over<br>PHQ-9 score ≥10 and/or GAD-7 score ≥8 (at any point during the therapy)<br>CAPE-P15 scores ≥1.47 for frequency and ≥1.47 for distress<br>Expressed an interest in participating in research either via the CAPE-P15 or IAPT registration form<br>Have completed or attended at least 4 therapy sessions at the time of recruitment	PHQ-9 score ≤10 and/or GAD-7 score ≤8<br>CAPE-P15 scores ≤1.47 for frequency and ≤1.47 for distress<br>Have not expressed an interest in participating in research neither in CAPE-P15 not IAPT registration form<br>Have attended fewer than 4 therapy sessions at the time of recruitment

For peer review only - http://bmjopen.bmj.com/site/about/guidelines.xhtml

Table 2: Inclusion and exclusion criteria for people who initiated treatment but withdrawn

Inclusion	Exclusion
Male or Female<br>18 years of age or over<br>PHQ-9 score ≥10 and/or GAD-7 score ≥8 (at any point during the therapy)<br>CAPE-P15 scores ≥1.47 for frequency and ≥1.47 for distress<br>Expressed an interest in participating in research either via the CAPE-P15 or IAPT registration form<br>Initiated treatment but withdrawn	PHQ-9 score ≤10 and/or GAD-7 score ≤8<br>CAPE-P15 scores ≤1.47 for frequency and ≤1.47 for distress<br>Have not expressed an interest in participating in research neither in CAPE-P15 nor IAPT registration form

Data collection

Semi-structured interviews will be conducted using a topic guide that is co-produced with our service user advisory group, and that is informed by a review of relevant literature. Members of our service-user advisory group and members of the research team developed the topic guide, and in so doing sought to minimize the risk of unduly stressing participants through content and delivery. The topic guide aims to elicit participants' views on the identification and treatment of people with a common mental disorder including psychotic experiences attending step-3 IAPT services. More specifically, it asks participants to discuss relevant aspects of their personal and life stories, including how past negative life events contributed to their mental health difficulties, and their positive and negative experiences of receiving care from IAPT services. The researcher will explore service users' views on what improvements might be made at the levels of the therapist and talking treatment. The interview will also examine how such improvements should be implemented and prioritized to improve the care that step-3 IAPT services deliver to people with a common mental disorder including psychotic experiences.

For peer review only - http://bmjopen.bmj.com/site/about/guidelines.xhtml

Recruitment, sampling, and selection of IAPT therapists and managers for interview

Our aim is to recruit five IAPT therapists and one manager per IAPT provider. We will present details of the study during team meetings and ask for volunteers, with more than six months experience working with step-3 IAPT service users. We will collect data on therapists/managers' years of professional practice, gender, ethnicity, and clinical orientation. We will use a purposive sampling method to ensure diversity of therapists and managers participants.

Data collection

Semi-structured interviews will be conducted using a topic guide which has been co-produced with an IAPT manager and also informed by a review of the relevant literature. The manager and members of the research team developed the topic guide collaboratively, with the aim to develop interview questions and prompts that covered the full scope of topics pertinent to the scope of the study. The topic guide aims to elicit participants' views on the identification and treatment of people with a common mental disorder including psychotic experiences attending step-3 IAPT services. More specifically, the topic guide will ask participants to discuss their positive and negative experiences of delivering care to service users with a common mental disorder including psychotic experiences, the talking therapies they use to treat and care for these users, and their reflections regarding effectiveness. The researcher will also explore the types of knowledge that participants draw upon to provide care and support to these service users. Additionally, the interview will ask about therapists' and managers' ideas, values and priorities for the development of a new talking treatment and for improving therapists' skills in treating service users with a common mental disorder including psychotic experiences.

For peer review only - http://bmjopen.bmj.com/site/about/guidelines.xhtml

Data analysis

All interview data will be coded by one researcher experienced in qualitative research methods. Although we will not formally test inter-rater reliability, a sample of the collected interview data (n=5) will be double-coded by another researcher. Analysis will be based on the constant comparative method as described by Charmaz. [36] We will draw on the sensitising constructs identified through the literature, but we will remain open to the emergence of other important themes which may be more relevant to the primary care mental health setting. NVivo software (NVivo v11, QSR International, Doncaster, Australia) will be used to facilitate data coding. The combination of the different strands of data (i.e. semi-structured interviews with IAPT users, therapists and managers), will be brought together to inform the development of a new talking therapy for people with a common mental disorder including psychotic experiences. More specifically, our qualitative study findings will inform the development of the talking therapy prior to its piloting in preparation for a randomised controlled trial (RCT) of the therapy. We will also use the findings to ensure that participants' priorities are incorporated in the theory of change of the intervention.

Limitations of the study

The study has several limitations. First, the expected findings may not be applicable to people younger than 18 years old. Second, the sample size will be small, so appropriate caution will be needed in the interpretation and generalisation of the findings. Third, the study will not include real-time observations of therapeutic encounters - which might generate more insight into how IAPT service users and therapists interact and co-construct care (i.e. access to enacted, not just espoused, valuations and preferences). Fourth, the recruitment and sampling methods may influence the expected findings, as service users who meet the inclusion criteria may not respond to our invitation due to the severity of their

For peer review only - http://bmjopen.bmj.com/site/about/guidelines.xhtml

mental health problems or they may not be selected to participate due to the limited capacity of the researcher to interview all participants who meet the inclusion criteria.

Patient and public involvement

The McPin Foundation (http://mcpin.org) convened a service user advisory group for the programme. This involves nine people with common mental disorders including psychotic experiences from diverse backgrounds, including ethnic minorities. For this particular work-package, the research team work collaboratively with the service user advisory group to develop the IAPT service user invitation letter, information sheet, and interview topic-guide list. All study participants will receive a digest summary of findings written in lay English language.

ETHICS AND DISSEMINATION

The following permissions have been obtained for this study:

1) NHS Ethics – approval granted by London Harrow Research Ethics Committee (reference: 18/LO/0642);
2) NHS Permissions were granted by all Trusts involved;
3) We registered the study on the UK Clinical Research Network Study Portfolio (reference: 38180)

Ethical considerations

A number of steps will be taken to ensure that the research is conducted ethically, protecting participants' rights and maintaining appropriate confidentiality of the information provided.

Voluntary nature of participation and right to withdraw

Information sheets for participants, as well as researcher verbal reassurance, will clarify that participation in the study is voluntary. No pressure will be placed on any

For peer review only - http://bmjopen.bmj.com/site/about/guidelines.xhtml

individual to take part in the research. It will be made clear to participants that their decision to take part or decline to take part in any part of the research will affect neither the care they receive nor their legal rights. Participants will also be informed about their right to withdraw from the study.

Informed consent

All participants will be asked to give their informed consent before any data are collected. Such consent will only be sought after they have been provided with full information about the research and what their participation would involve. Additionally they will be given sufficient time to consider the aims and scope of the study and ask questions. Service users will not be approached about the research if – in the opinion of mental health professionals – (i) they are unable to give informed consent, (ii) their current mental health gives significant cause for concern, and (iii) their mental health may be adversely affected by taking part in the research. The researcher will liaise with the participating IAPT teams to ensure that the potential participants selected to be contacted and invited to participate in an interview do not meet any of the above criteria.

Safety of participants and researcher

Consideration has been given to ways in which taking part in the research might be harmful to participants (in particular service users) and steps will be taken to manage such potential outcomes should they occur. Clinical risk assessments are routinely undertaken within IAPT services to identify during the initial assessment anyone at high risk of harm to themselves or to other people. High risk individuals will be referred on to appropriate services and, accordingly, not be included within our sample. Nevertheless, reflecting on mental health, traumatic experiences and events, and past health care experiences may cause distress and measures will be taken to ensure the safety of participants. Should participant distress occur during the interviews the researcher will pause the interview, and support the

For peer review only - http://bmjopen.bmj.com/site/about/guidelines.xhtml

participant to manage the distress and regulate their emotions. The researcher will then check if s/he is still willing to continue with the interview process. If a participant remains distressed, the researcher will ask for permission to contact the participant's GP or mental health worker, whilst, encouraging the participant to seek further support that may be available in their environment. The researcher will have up-to-date contact information for psychiatric emergency services, if required. This will include access to First Response Services and/or Crisis Resolution Home Treatment Teams. These services should be able to advise about how to manage difficult clinical scenarios.

If, during the interview, a participant suggests that they intend to harm themselves or another person, the researcher will ask for the contact details of the participant's GP and inform their GP or mental health worker. The researcher will inform the participant about this action and the reasons for doing this. As stated above, the researcher might also contact psychiatric emergency services in the area. This limit to confidentiality will be explicit in the research information sheet, and it will be explained again before the start of the interview. We believe that the risk of psychological distress to the IAPT therapists and managers will be minimal.

Consideration has also been given to the safety of the researcher who will carry out the interviews. The researcher has attended training to equip him with the skills, awareness and knowledge required for the safe conduct of research in community settings. He is also experienced in carrying out research with vulnerable individuals, including offenders, homeless people, and people with severe mental illness. The researcher will follow the THIS Institute's (The Healthcare Improvement Studies Institute, www.thisinstitute.cam.ac.uk) lone worker policy when interviewing people at their homes.

For peer review only

For peer review only - http://bmjopen.bmj.com/site/about/guidelines.xhtml

Dissemination

We will report the findings to national and international, multi-disciplinary audiences to inform future research and practice using a variety of methods. We will disseminate original articles in peer-reviewed academic journals, conference presentations, and learning reports. We will also produce a "digest" summary of the findings, which will be accessible, visual, and freely available.

STUDY STATUS

Data collection started in June 2018 and is expected to last 7 months.

DISCUSSION

Improving the prospects of recovery for all people experiencing mental health disorders is a priority in the mental health policy agenda in England. Within this context, this study aims to inform the development of a new talking therapy that will increase the prospects of recovery for people with a common mental disorder including psychotic experiences.

AUTHORS' CONTRIBUTION

Conception or design of the work

Peter B. Jones, Jesus Perez, Robbie Duschinsky, Mary Dixon-Woods

Drafting the article

Alexandros Georgiadis, Robbie Duschinsky, Mary Dixon-Woods

Critical revision of the article

Alexandros Georgiadis, Robbie Duschinsky, Jesus Perez, Peter B. Jones, Debra Russo, Clare Knight, Emma Soneson, Mary Dixon-Woods

For peer review only - http://bmjopen.bmj.com/site/about/guidelines.xhtml

Final approval of the version to be published

Alexandros Georgiadis, Robbie Duschinsky, Jesus Perez, Peter B. Jones, Debra Russo, Clare Knight, Emma Soneson, Mary Dixon-Woods.

ACKOWLEDGMENTS

The authors would like to thank the McPin Foundation, the TYPPEX Lived Experience Advisory Panel, and the participating research sites for helping us to set up this research.

FUNDING STATEMENT

This work was supported by the National Institute for Health Research grant number RP-PG-0616-20003. This work was also supported by Mary Dixon-Wood's Wellcome Trust Investigator award WT09789 and by THIS Institute, which is supported by the Health Foundation. Mary Dixon-Woods and Peter Jones (emeritus) are National Institute for Health Research (NIHR) Senior Investigators.

DECLARATION

This report is independent research funded by the National Institute for Health Research (Programme Grants for Applied Research, Tailoring evidence-based psychological therapY for People with common mental disorder including Psychotic EXperiences (TYPPEX), RP-PG-0616-20003). The views expressed in this publication are those of the author(s) and not necessarily those of the NHS, the National Institute for Health Research or the Department of Health.

COMPETING INTERESTS

The authors declare no competing interests.

WORD COUNT: 3714

REFERENCES

1. Hui C, Morcillo C, A RD, et al. Psychiatric morbidity, functioning and quality of life in young people at clinical high risk for psychosis. *Schizophrenia Bulletin* 2013;148(1-3):175-80.
2. Fusar-Poli P, Nelson B, Valmaggia L, et al. Comorbid depressive and anxiety disorders in 509 Individuals with an at-risk mental State: Impact on psychopathology and transition to psychosis. *Schizophrenia Bulletin* 2014;40(1):120-31.
3. Kelleher I, Lynch F, Harley M, et al. Psychotic symptoms in adolescence index risk for suicidal behavior: findings from 2 population-based case-control clinical interview studies. *Archives in General Psychiatry* 2012;69(12):1277-83.
4. Hutton P, Bowe S, Parker S, et al. Prevalence of suicide risk factors in people at ultra-high risk of developing psychosis: a service audit. *Early Intervention in Psychiatry* 2011;5(4):375-80.
5. Granö N, Karjalainen M, Souminen K, et al. Poor functioning ability is associated with high risk of developing psychosis in adolescents *Nordic Juornal of Psychiatry* 2011;65(1):16-21.

For peer review only - http://bmjopen.bmj.com/site/about/guidelines.xhtml

For peer review only

6. Salokangas RK, Ruhrmann S, von Reventlow HG, et al. Axis I diagnoses and transition to psychosis in clinical high-risk patients EPOS project: prospective follow-up of 245 clinical high-risk outpatients in four countries. *Schizophrenia Bulletin* 2012;138(2-3):192-97.
7. Wigman JT, van Nierop M, Vollebergh WAM, et al. Evidence that psychotic symptoms are prevalent in disorders of anxiety and depression, impacting on ilness onset, risk, and severity - implication for diagnosis and ultra-high risk research. *Schizophrenia Bulletin* 2012;38(2):247-57. doi: 10.1093/schbul/sbr196
8. Wigman J, van Os J, Abidi L, et al. Subclinical psychotic experiences and bipolar spectrum features in depression: association with outcome of psychotherapy. *Psychological Medicine* 2014;44(2):325-36.
9. Perlis RH, Uher R, Ostacher M, et al. Association between bipolar spectrum features and treatment outcomes in outpatients with major depressive disorder. *Archives in General Psychiatry* 2011;68(4):351-60.
10. Are psychotic features in adolescents with depression a predictor of severity? Congress of Psychiatry Medforum; 2015; Wisla, Poland.
11. Kelleher I, Keeley H, Corcoran P, et al. Clinicopathological significance of psychotic experiences in non-psychotic young people: evidence from four population-based studies. *British Journal of Psychiatry* 2012;1:26-32.
12. Varghese D, Scott J, Welham J, et al. Psychotic-like experiences in major depression and anxiety disorders: A population-based survey in young adults. *Schizophrenia Bulletin* 2009;37(2):389-93.
13. Lichtenstein P, Yip BH, Bjork C, et al. Common genetic determinants of schizophrenia and bipolar disorder in Swedish families: a population-based study. *Lancet* 2009;17(373):234-39.
14. van Os J, Linscott R, Myin-Germeys I, et al. A systematic review and meta-analysis of the psychosis continuum: evidence for a psychosis proneness-persistence-impairment model of psychotic disorder. *Psychological Medicine* 2009;39(2):179-85.
15. Nishida A, Tanii H, Nishimura Y, et al. Associations between psychotic-like experiences and mental health status and other psychopathologies among Japanese early teens. *Schizophrenia Bulletin* 2008;99(1-3):125-33.
16. Kraan T, Velthorst E, Smit F, et al. Trauma and recent life events in individuals at ultra high risk for psychosis: Review and meta-analysis. *Schizophrenia Bulletin* 2015;161(2-3):143-49.
17. Russo DA, Stochl J, Painter M, et al. Trauma history characteristics associated with mental states at clinical high risk for psychosis. *Psychiatric Research* 2014;220(1-2):237-44.
18. Kelleher I, Keeley H, Corcoran P, et al. Childhood trauma and psychosis in a prospective cohort study: cause, effect and directionality. *American Journal of Psychiatry* 2013;170(7):734-41.
19. National Collaboration Centre for Mental Health. The Improving Access to Psychological Therapies Manual. London, 2018.
20. Clark DM, Canvin L, Green J, et al. Transparency about the outcomes of mental health services (IAPT approach): an analysis of public data. *Lancet* 2018;391:679-86.
21. Kroenke K, Spitzer RL. The PHQ-9: a new depression diagnostic and severity measure. *Psychiatric Annals* 2002;32:509-15.
22. Spitzer RL, Kroenke K, Williams JB, et al. A brief measure for assessing generalised anxiety disorder: the GAD-7. *Archives of Internal Medicine* 2006;166:1092-97.
23. NHS Digital. Psychological Therapies: Annual report on the use of IAPT services England, further analyses on 2016-17. . Leeds, 2017.
24. Perez J, Russo DA, Stochl J, et al. Common mental disorder including psychotic experiences: Trailblaizing a new recovery pathway within the Improving Access to Psychological Therapies programme in England. *Early Intervention in Psychiatry* 2017:1-8. doi: 10.1111/eip.12434
25. British Medical Association. Breaking down barriers - the challenge of improving mental health outcomes London: British Medical Association, 2017.

For peer review only - http://bmjopen.bmj.com/site/about/guidelines.xhtml

26. Dunston R, Lee A, Boud D, et al. Co-production and health system reform - From re-Imagining to re-Making. *Australian Journal of Public Administration* 2009;68(1):39-52. doi: 10.1111/j.1467-8500.2008.00608.x
27. Batalden M, Batalden P, Margolis P, et al. Coproduction of healthcare service. *BMJ Quality & Safety* 2015;25:509-17.
28. Sabadosa KA, Batalden PB. The interdependent roles of patients, families, and professionals in cyctic fibrosis: a system for the coproduction of healthcare and its improvement. *BMJ Quality & Safety* 2014;23:i90-94.
29. Slay J, Stephens L. Co-production in mental health: a literature review. London: New Economics Foundation 2013.
30. Biringer E, Hartveit M, Sundfor B, et al. Continuity of care as experienced by mental health service users - a qualitative study. *BMC Health Services Research* 2017;17(763) doi: https://doi.org/10.1186/s12913-017-2719-9
31. Clark M. Co-production in mental health care. *Mental Health Review Journal* 2015;20(4)
32. Memon A, Taylor K, Mohebati ML, et al. Perceived barriers to accessing mental health services among black and minority ethinc (BME) communities: a qualitative study in Southeast England. *BMJ Open* 2016;6 doi: 10.1136/bmjopen-2016-012337
33. Bee P, Brooks H, Fraser C, et al. Professional perspectives on service user and care involvement in mental health care planning: A qualitative study. *International Journal of Nursing Studies* 2015;52(12):1834-45. doi: https://doi.org/10.1016/j.ijnurstu.2015.07.008
34. Crotty M. The Foundations of Social Research: Meaning and Perspective in the Research Process. London: Sage Publications 1998.
35. Forman J, Creswell JW, Damschroder L, et al. Qualitative research methods: Key features and insights gained from use in infection prevention research *Amrican Journal of Infection Control* 2008;36(10):764-71. doi: https://doi.org/10.1016/j.ajic.2008.03.010
36. Charmaz K. Constructing Grounded Theory. Thousand Oaks, California: Sage 2014.
37. Capra C, Kavanagh DJ, Hides L, et al. Current CAPE-15: a measure of recent psychotic-like experiences and associated distress. *Early Intervention in Psychiatry* 2013;11(5):411-17. doi: 10.1111/eip.12245
38. Bukenaite A, Stochl J, Mossaheb N, et al. Usefulness of the CAPE-P15 for detecting people at ultra-high risk for psychosis: Psychometric properties and cut-off values. *Schizophrenia Research* 2017;189:6-74.

For peer review only - http://bmjopen.bmj.com/site/about/guidelines.xhtml
